# Examining Associations Between Sociodemographic Characteristics and Ever Breastfed Children, NHANES 1999–2020

**DOI:** 10.3390/ijerph22030428

**Published:** 2025-03-14

**Authors:** Jessica Amezcua, Lindsey M. West, Camelia Malkami, Marlo Vernon, Elinita Pollard, Justin X. Moore

**Affiliations:** 1 Markey Cancer Center, University of Kentucky, Lexington, KY 40536, USA; jessica.amezcua@uky.edu (J.A.); elinitapollard@uky.edu (E.P.); 2 Department of Psychiatry and Health Behavior, Medical College of Georgia, Augusta University, Augusta, GA 30912, USA; liwest@augusta.edu; 3 Medical College of Georgia, Augusta University, Augusta, GA 30912, USA; cmalkami@augusta.edu; 4 Department of Medicine, Medical College of Georgia, Augusta University, Augusta, GA 30912, USA; mvernon@augusta.edu; 5 Center for Health Equity Transformation, Department of Behavioral Science, School of Medicine, University of Kentucky, Lexington, KY 40536, USA

**Keywords:** breastfeeding, health disparities, NHB mothers, poverty–income ratio

## Abstract

Although breastfeeding provides health benefits to both mother and child, this study aimed to explore whether disparities in breastfeeding continue to exist, particularly among non-Hispanic Black (NHB) mothers and children. We performed a cross-sectional analysis among 19,830 children in the United States (US) using the National Health and Nutrition Examination Survey (NHANES) data from 1999 to 2020. Breastfeeding initiation and duration rates increased overall from 1999 to 2020. Children who were ever breastfed were more likely to have higher body weight, older mothers, mothers who did not smoke during pregnancy, a higher family poverty–income ratio (PIR), food security, mothers in excellent health, and mothers who had not seen a mental health professional in the past year. NHB children were breastfed at significantly lower rates and for shorter duration than non-Hispanic White (NHW), Mexican, Other Hispanic, and Other/Multi-Racial children. NHB children were breastfed less than other racial groups, including minority Mexican children with similar average PIR, suggesting a possible unique experience for NHB mothers and children. Strategies include impacting social norms and offering culturally tailored breastfeeding supports. The provision of structural supports to remove barriers to breastfeeding is a social justice issue. Breastfeeding confers health benefits to mother and child, and disparities exist among mothers and children, particularly among NHB mothers and children. The current study provides data on the most recent breastfeeding trends, showing that these disparities by race/ethnicity are present. Interestingly, even among Mexican participants of a similar PIR, NHB children were still breastfed less.

## 1. Introduction

### 1.1. Breastfeeding Health Benefits and Risk Reduction for Mothers and Infants

The World Health Organization recommends exclusive breastfeeding for infants for the first 6 months of life, and then, with the introduction of solid foods, for breastfeeding to continue to at least 2 years of age, with increased benefits up to 12 months, and even up to 24 months [1]. Breastfeeding beyond 12 months provides infants increased protection from infectious diseases and against hypertension, metabolic syndrome, cardiovascular disease, and possibly osteoporosis [2].

Breastmilk reduces the risk for asthma, obesity, Type 1 diabetes, severe lower respiratory disease, acute otitis media, sudden infant death syndrome (SIDS), gastrointestinal infections, atopic dermatitis, gastrointestinal infection, lower respiratory infection, acute lymphocytic leukemia, acute myelogenous leukemia, and necrotizing enterocolitis [3,4,5,6]. Breastfeeding is significantly associated with reduced post-perinatal infant deaths among different racial and ethnic groups in the United States (US) [4]. Further, breastfed children have lower incidence of obesity and Type 2 diabetes later in life [6]. Breastfeeding reduces the mother’s risk for hypertension, Type 2 diabetes, ovarian cancer, and breast cancer [7]. There would also be a $13-billion per year savings in health care costs if 90% of mothers breastfed their infants exclusively for 6 months [8]. These cost savings are particularly revealing given the costs associated with breastfeeding (e.g., pumping supplies).

### 1.2. Ever Breastfed Rates in the United States

Since 2014, there has been a slight increase in breastfeeding initiation rates. Among infants born in 2021, 84.1% began receiving some breast milk, and, by six months, 59.8% were still being breastfed [9]. Exclusive breastfeeding rates in 2021 were 46.5% through three months and 27.2% through six months, showing little change since 2014 [9]. Currently, Healthy People 2030 aims to increase the percentage of infants exclusively breastfed for six months to 42.9% [10].

### 1.3. Sociodemographic Factors and Breastfeeding

According to the CDC, from 1 January 2020 to 31 December 2021, exclusive breastfeeding rates by race/ethnicity revealed the highest rates of ever attempting breastfeeding among Asian American women (90.3%), followed by Hispanic women (86.8%), non-Hispanic White (NHW) (85.9%), multiracial women (83.1%), then Asian American/Pacific Islander women (79.4%), Native American/Alaska Native women, (77.7%), and non-Hispanic Black women (NHB) (74.5%) [11,12]. The most recent breastfeeding duration rates reported by the CDC from 2011 to 2015 show a decline at the 6-month and 12-month age period across all racial groups. Rates ranged from 47.8% to 73.5% at 6 months and from 26.1% to 50.0% at 12 months, with NHB women consistently showing the lowest adherence and Asian women the highest adherence over both time periods [12,13]. Other sociodemographic variables from this same cohort revealed the highest rates of breastfeeding adherence among college graduates (93.9%), women aged 30 years or older (85.2%), those with the highest incomes (93.1%), married women (89.7%), those living in a metropolitan area (84.9%), being first born (85%), and those ineligible for the Special Supplemental Nutrition Program for Women, Infants, and Children (WIC) (92.1%) [13].

Data from the National Health and Examination Survey (NHANES), representing 2069 participants from 2009 to 2014, revealed that breastfeeding rates differed significantly among NHW women, Hispanic women, and NHB women at 80.0%, 77.5%, and 57.4%, respectively (*p* < 0.001), even when controlling for age, education, and income [14]. A study examining NHANES 2003 to 2012 found that Mexican and NHB mothers were most likely to cease breastfeeding and that their infants and toddlers were more likely to be overweight [15]. The National Survey of Children’s Health 2011 and 2012 data of 26,000 caregivers found that single-parent and step-families, lower-income families, NHB children, children with exposure to tobacco, and children of mothers with lower education are at the greatest risk of not initiating breastfeeding or at a risk of less-than-optimal breastfeeding duration [16].

Cultural factors could also increase breastfeeding rates. For example, a study by Gibson et al. (2005) examined the relationship between acculturation status (i.e., identifying/borrowing cultural traditions from a culture most represented where one resides) and breastfeeding among Hispanic women residing in the United States, using NHANES data from 1999 to 2000 [17]. Acculturation was defined by language spoken at home, country of birth, language ability, and country of last schooling. The study found that breastfeeding prevalence was higher among less acculturated Hispanic women (59.2%) compared to highly acculturated Hispanic women (33.1%) and NHW women (45.1%) [17]. In other words, Hispanic women who identified more closely with their Hispanic cultural values and traditions were more likely to breastfeed and less influenced by the cultural norms of the dominant society. Less acculturated Hispanic women were more likely to cite their child’s physical/medical condition as a reason not to breastfeed (53.1%), whereas NHW parents and more acculturated Hispanic women were more likely to cite their child preferred the bottle (57.5% and 49.8%) [17]. Likewise, a meta-ethnography found the “maternal culture” or beliefs and knowledge surrounding breastfeeding can serve as a facilitator or barrier to breastfeeding uptake [18]. Among NHB women, subjective norms were the strongest predictors of exclusive breastfeeding intention, with family and community shaping these norms [19].

NHB mothers, regardless of income and education, are breastfeeding less than any other group [20]. Further, NHB women are more likely to die from pregnancy-related complications than other racial groups; the maternal mortality rate for NHB women is two to four times higher than it is for NHW women [21], making breastfeeding impossible for a larger proportion of NHB infants. Moreover, NHB mothers are nine times more likely to be offered formula at the hospitals and providers are less likely to discuss breastfeeding with NHB mothers than NHW women [22,23,24]. Thus, access to care may not be as strong of a facilitator of breastfeeding for NHB women. The current study aims to expand on previous NHANES studies by exploring sociodemographic characteristics that may explain differences in breastfeeding in a nationally representative sample of ever breastfed infants and toddlers.

## 2. Methods

### 2.1. Research Design

#### 2.1.1. Setting and Relevant Context

We performed a cross-sectional analysis using data from NHANES, a nationally representative sample of non-institutionalized US residents. We examined trends in breastfeeding using NHANES data collected from 1999 through March 2020. NHANES interviewers asked questions related to infant nutrition and breastfeeding to proxy respondents only for children six years of age and younger. Because of this, our study population was restricted to children aged zero to six years, as breastfeeding-related questions were limited to this group. Although, the study population consisted of children aged zero to six, the data were gathered through the responses of mothers and caregivers.

#### 2.1.2. Sample

The NHANES study, conducted by the National Center for Health Statistics (NCHS) of the Centers for Disease Control and Prevention (CDC), is an ongoing program designed to assess the health and nutritional status of adults and children in the United States. Since its inception in 1959, NHANES has utilized a stratified, multistage probability sampling design to survey a nationally representative sample of approximately 5000 participants annually [25]. To ensure the adequate representation of key subpopulations, the survey oversamples individuals aged 60 and older, Mexican Americans, and non-Hispanic Black individuals. NHANES collects a wide range of data, including demographic, socioeconomic, dietary, and health-related information, along with physical examinations and laboratory measures. For this study, data from NHANES 1999 to 2020 were analyzed. Inclusion criteria were survey responses for NHANES participants aged 0 to 6 years. After applying these criteria, a final analytical sample of 19,830 participants over the 21-year study period was included. All NHANES data collection procedures were approved by the NCHS Research Ethics Review Board, and participants provided informed consent prior to participation.

#### 2.1.3. Measurement

Our primary outcome of interest was whether a child was ever breastfed. From the NHANES survey questionnaire, breastfeeding initiation and duration status were determined using the following questions: (1) “Was the sample person (infant/toddler) ever breastfed or fed breast milk?” and (2) “How old was the sample person (infant/toddler) when (he or she) completely stopped breastfeeding or being fed breast milk?” Breastfeeding initiation (henceforth, ever or never breastfed) was classified as a binary measure (yes/no). The age of discontinued breastfeeding was recorded as a continuous measure (in days) and categorized into four groups: 0–6 weeks, 6 weeks–6 months, 6 months–1 year, and greater than 1 year. The categorization of age was based on commonly recognized milestones in breastfeeding practices and recommendations. These categories reflect different stages of infant and child development. The American Academy of Pediatrics (APP) recommends exclusive breastfeeding for the first 6 months, followed by continued breastfeeding for at least 1 year or longer [26].

Socio-demographic characteristics included in this study were sex at birth, race/ethnicity (classified as non-Hispanic White, non-Hispanic Black, Mexican American, Other Hispanic, and Other or Multiracial), birthweight (continuous, in pounds and ounces), mother’s age at childbirth (continuous), mother’s smoking status during pregnancy (yes/no/missing), citizenship status (US citizen or not), insurance coverage (reported as insured/uninsured at the time of the survey or missing), household food security status (measured using the US 18-item Household Food Security Survey Module and categorized as full food security, marginal, low, very low food security, or missing), and family poverty–income ratio (PIR). PIR is a measure of total family income relative to the federal poverty threshold. It is calculated solely based on reported family income and the corresponding national poverty threshold, without taking regional cost-of-living differences into account. Individuals who reported no income were assigned a PIR value of zero. A PIR of less than 1 indicates income below the official poverty line, values above 1 reflect income levels above the poverty line, and values approaching 5 represent households with significantly higher income levels. Additional measures included mothers’ self-reported general health condition and whether the mother had seen a mental health professional in the past year (yes/no).

#### 2.1.4. Data Collection

Operated by the CDC’s NCHS, NHANES is a stratified, multistage, cross-sectional survey designed to assess the health and nutritional status of a nationally representative sample of U.S. residents, including seniors, adults, pregnant women, and children, including infants and toddlers. For this study, proxies of the children aged 0–6 years completed a household interview and health examination components. All data collection procedures for NHANES were conducted by trained interviewers using standardized protocols, with interviews administered in person at mobile examination centers or during home visits. Detailed protocols for NHANES data collection are available through the handbook protocol [25]. All responses were self-reported, with data quality ensured through rigorous interviewer training and validation procedures.

#### 2.1.5. Data Analysis

Appropriate complex survey design sample weights were considered to account for the probabilities of selection for the participants, oversampling, and missing information in the NHANES survey. Refused responses to questions or questions that were unknown were categorized as missing. In our analysis, we evaluated the extent of missing data for all key variables. For example, the proportion of missing data was [257 participants or 1.3% of 13,762 that had ever breastfed] for breastfeeding duration and ranged from [56.98% for mother’s seeing a mental health professional] to [0%] for socio-demographic covariates such as mother’s age (0.3%, unweighted), family PIR (8.6%, unweighted), household food security (2.8%, unweighted), and insurance coverage (0.58%, unweighted). Based on our assessment, the missingness appeared to be missing completely at random (MCAR)/missing at random (MAR). We conducted descriptive analyses and reported survey-weighted means and SDs for continuous variables, and frequencies and percentages for categorical variables. The Rao–Scott weighted chi-square test was used to assess statistical difference in food insecurity prevalence by sociodemographic for categorical variables, while weighted F-tests were used for continuous variables. We estimated the prevalence of breastfeeding from weighted frequency and relative frequencies by year, year and race/ethnicity, and year and age at breastfed discontinued. PIR was calculated as the ratio of total family income to poverty threshold values (in dollars) and adjusted for inflation. People without income were assigned a zero value for PIR. PIR values less than one are considered below the official poverty line, whereas PIR values greater than one are above the poverty level [27]. Household-level information, such as PIR and food security status, was collected during the in-home interview from an adult member who was aged ≥18 years, had a significant role in household management, and was designated the household reference person. This reference person generally referred to as the primary caregiver/parent, also answered questions about feeding practices at the in-home interview.

The primary outcome of interest was the prevalence of ever being breastfed. We performed several weighted and modified Poisson regression models to predict ever breastfed status between race/ethnicity groups, adjusting for potential confounders, including mother’s age, family PIR, citizenship status, insurance coverage, household food security, and mother smoking status during pregnancy. Estimates derived from weighted Poisson regression are presented as prevalence ratios (PRs) and associated 95% confidence intervals (CIs). All statistical analyses were conducted using the SAS statistical software package (version 9.4, SAS Institute, Inc., Cary, NC, USA, 2019). *p* values < 0.05 were considered statistically significant.

## 3. Results

**Figure 1:**
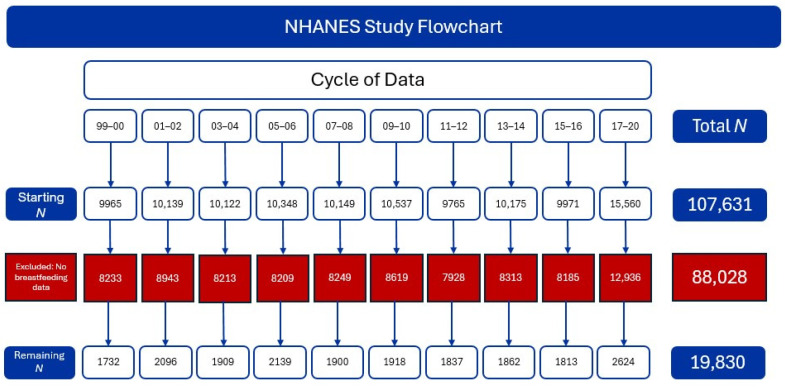
Flowchart of NHANES study participants by each cycle and exclusion criteria.

Our analytic population consisted of 19,830 NHANES participants, representing 27,883,398 US children’s mothers surveyed between 1999 and March 2020 (Figure 1).

Table 1 displays the descriptive comparisons between ever breastfed and never breastfed children for several demographic characteristics. On average, breastfed children weighed more at birth than children never breastfed (7.34 pounds vs. 7.05; *p*-value < 0.0001). In general, mothers of children that were breastfed were older (28.4 vs. 26.0 years old; *p*-value < 0.0001), less likely to smoke while pregnant (90.8% vs. 76.2%; *p*-value < 0.0001), and more likely to not be US citizens (1.8% vs. 0.6%; *p*-value < 0.0001), have a higher income (Mean PIR = 2.52 vs. 1.82, *p*-value < 0.0001), have full food security (70.4% vs. 63.2%; *p*-value < 0.0001), report excellent health condition (60.0% vs. 52.0%; *p*-value < 0.0001), and not have seen a mental health professional in the past year (96.0% vs. 94.4%; *p*-value = 0.0141) when compared to mothers that did not breastfeed. There were no significant differences in insurance status and sex in breastfeeding status of children.

**Figure 2:**
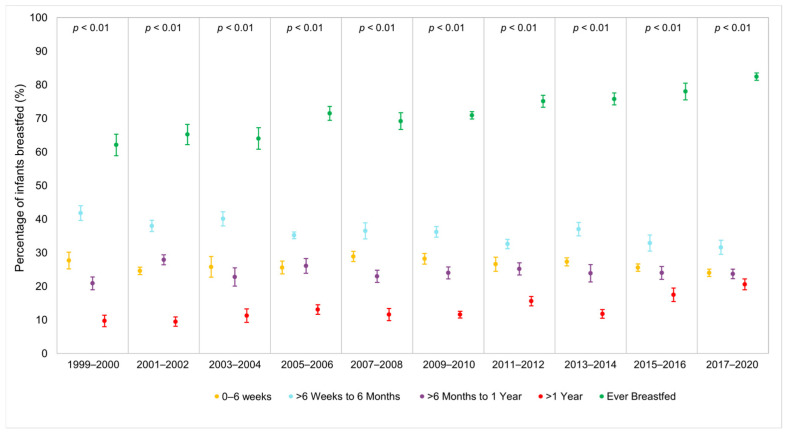
Percentage of infants ever breastfed, stratified by duration breastfed in NHANES cohort: United States, 1999–2020.

**Figure 3:**
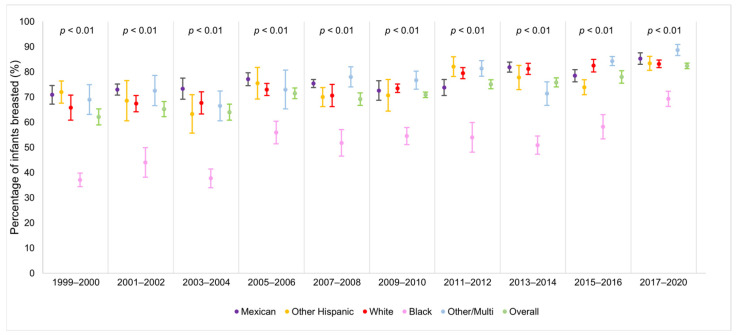
Percentage of infants who were breastfed by NHANES cohort and race–ethnicity: United States 1999–2020.

**Figure 4:**
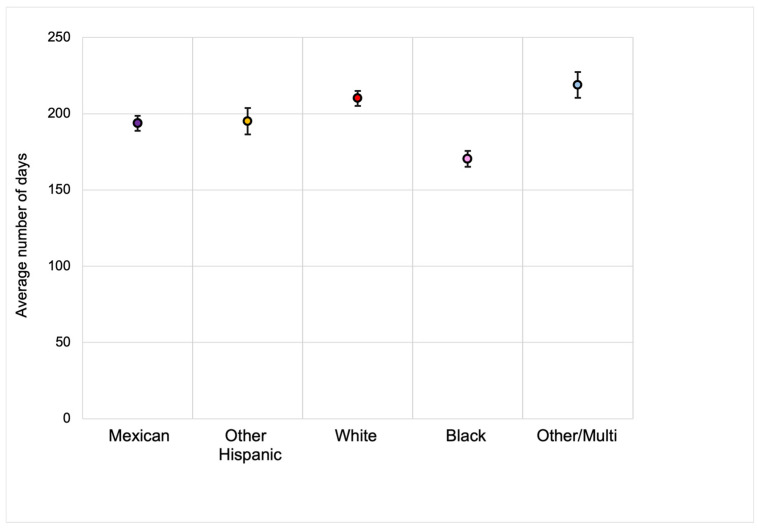
Average number of days breastfed by race-ethnicity: United States, 1999–2020.

**Figure 5:**
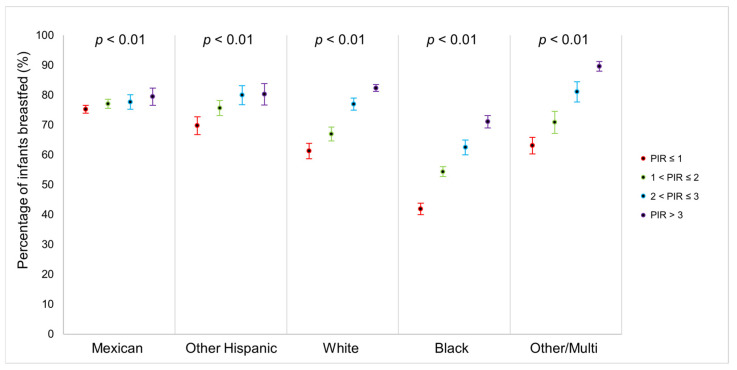
Percentage of infants who were ever breastfed by PIR and race–ethnicity: United States, 1999–2020.

We observed an upward trend in the total proportion of children aged 0–6 years, who were ever breastfed from 1999 to March 2020 (Figure 2; Supplementary Table S1). When examining breastfeeding rates by race and ethnicity, a striking pattern emerged: NHB children experienced the lowest breastfeeding rates but showed the greatest increase over time, rising from 37.1% in 1999–2000 to 69.3% in 2017–2020 (Figure 3; Supplementary Table S2). However, significant racial and ethnic differences were observed in breastfeeding duration. As shown in Figure 4 and Supplementary Table S3, NHB children were breastfed the shortest duration, with an average of 170.38 days (95% CI = 160.14–180.62). In contrast, NHW children were breastfed for an average of 210.14 (95% CI = 200.55–219.74). Overall, children across all racial and ethnic groups were breastfed for an average of 202.98 days (95% CI = 196.39–209.57). Regarding poverty–income ratio (PIR), NHW children lived in households with a higher PIR (PIR = 2.81, 95% CI = 2.70–2.91) compared to both NHB children (PIR = 1.52, 95% CI = 1.44–1.60) and Mexican children (PIR = 1.48, 95% CI = 1.42–1.54), as shown in Figure 5 and Supplementary Table S4. While PIRs were similar between NHB and Mexican children, breastfeeding rates varied according to PIR levels among NHB children (41.9–71.1%) but remained consistently high among Mexican children (75.3–79.5%). Furthermore, NHB children were less likely to have been breastfed in households with lower PIR.

Based on these data, breastfeeding rates for NHB children are likely to continue increasing, especially with ongoing interventions. However, breastfeeding rates for Mexican children may decrease due acculturation-related factors. To sustain or further increase breastfeeding rates, continued and targeted interventions are necessary.

### Regression Analysis

In unadjusted models, NHB children were 20% less likely to have been breastfed (PR = 0.80, 95% CI = 0.79–0.82) compared with NHW children (Table 2). Mothers with younger age were less likely to breastfeed their children; mothers aged 20 and under were 22% less likely (PR = 0.78, 95% CI = 0.76–0.80) and mothers aged 21–25 were 13% less likely (PR = 0.87, 95% CI = 0.85–0.89) to breastfeed when compared to mothers aged 31–35 years of age. Mothers with lower income, including those with PIR < 1 (PR = 0.81, 95% CI = 0.80–0.83), PIR between 1 and 2 (PR = 0.87, 95% CI = 0.85–0.89), and those with PIR between 2 and 3 (PR = 0.94, 95% CI = 0.92–0.96), had 19%, 13%, and 6% lower prevalence of ever breastfeeding their children when compared with mothers with PIR greater 3. Mothers with US citizenship were 16% less likely to have ever breastfed their children when compared to mothers with non-US citizenship (PR = 0.84, 95% CI = 0.79–0.88). Greater food insecurity was associated with lower likelihood of mothers breastfeeding their children, as mothers living with very low food security were 8% less likely to have ever breastfed their children (PR = 0.92, 95% CI = 0.89–0.95). Mothers who smoked while pregnant were 22% less likely to have ever breastfed their children (PR = 0.78, 95% CI = 0.76–0.80).

After multivariable adjustments, we observed that NHB children were still 16% less likely to have been breastfed (Model 3 adjusted PR = 0.84, 95% CI = 0.82–0.86) when compared to NHW children. Mexican children were 5% more likely to have been breastfed (Model 3 adjusted PR = 1.05, 95% CI = 1.03–1.07) when compared to NHW children. Still after adjustments, mothers who were younger, had lower income, held US citizenship, and smoked during pregnancy were significantly less likely to have ever breastfed their children. The effect of food security attenuated after adjusting for other sociodemographic characteristics and was not statistically associated with ever breastfeeding children.

## 4. Discussion

Breastfeeding initiation and duration rates increased from 1999 to 2020 in this study. Ever breastfed children were more likely to weigh more, have older mothers, have mothers who did not smoke during pregnancy, have higher PIRs, be unaffected by food insecurity, be in excellent health condition, and not have seen a mental health professional in the past year. NHB children were breastfed at significantly lower rates and for shorter duration than NHW, Mexican, Other Hispanic, and Other/Multi-Racial counterparts. Interestingly, there was no significant relationship between insurance status and breastfeeding status despite the Patient Protection Affordable Care Act (PPACA) being enacted during this study’s data collection. PPACA requires employers to guarantee employees time for breastfeeding and insurance companies to provide breastfeeding supplies and support services [28].

In our study we examined an upward trend in breastfeeding initiation and duration in the 21.3-year period examined, most notably for NHB women, highlighting significant racial and socioeconomic disparities in breastfeeding. We observed significant race-/ethnicity-specific interactions between PIR and breastfeeding initiation, with notably lower breastfeeding rates among NHB children living in lower-PIR households. Although NHB and Mexican mothers had a similar average PIR and age at birth in our study, NHB children were still the least likely to be breastfed, while Mexican children were among the most likely. In fact, Mexican children were breastfed at consistently high rates (75.3–79.5%) across all PIR levels, while breastfeeding rates among NHB children ranged from 41.9% at a PIR of less than or equal to 1, increasing to 71.1% at a PIR greater than 3. When looking at breastfeeding duration rates, the results showed that NHB children were breastfed for a significantly shorter duration of time than NHW and Mexican children. These disparities in breastfeeding are particularly concerning when considering that, for NHB women, the maternal mortality rate has increased by more than twofold since 1999, reaching 68 deaths per 100,000 live births in 2019 compared to only 27.9 deaths per 100,000 live births among NHW women [21]. This highlights the compounded challenges faced by NHB mothers, where socioeconomic status serves as a significant effect modifier for breastfeeding outcomes.

Consistent with studies utilizing NHANES data, our study also revealed differences in feeding practices [15]. Mothers’ physical health was closely related to breastfeeding, with previous research showing that biological or psychological difficulties with breastfeeding may also result from maternal overweight or obesity [29]. Other conditions, such as mastitis and postpartum depression, also act as barriers to breastfeeding [30,31]. Since breastfeeding disparities continue to exist regardless of sociodemographic factors, there must be structural factors inhibiting NHB, Indigenous, and People of Color (BIPOC) mothers from breastfeeding. One structural factor that may be contributing to this difference could be the focus on NHW mothers and babies and not BIPOC mothers and babies [24].

When considering factors that contribute to reduced breastfeeding rates among NHB mothers, it is crucial to consider the historical context of breastfeeding within this community. Generational trauma as a result of wet-nursing during slavery may serve as a barrier to breastfeeding among NHB mothers [32]. Over the years, grassroots organizations have been on the frontlines of this health crisis. In the early 2000s, the African American Breastfeeding Alliance was founded by four African American healthcare professionals as one of the first initiatives aimed to increase education and representation of NHB women in breastfeeding, and counter the messaging that encouraged to prioritize formula [33]. Following this, national organizations like the Black Mamas Matter Alliance were established to provide greater breastfeeding support and highlight unethical marketing practices targeting economically disadvantaged families and racial minorities [34]. To further address culturally relevant factors, the Black Mothers Breastfeeding Association and Black Breastfeeding Week were established in 2012 and 2013, aiming to reduce racial disparities, celebrate, raise awareness, and provide support for NHB mothers and families. Partner support has also proven essential in breastfeeding success, with NHB women noting that emotional, informational, and instrumental support from their partners played a vital role in both initiating and continuing breastfeeding [35]. Additionally, social media breastfeeding support groups have proven effective in boosting confidence, and self-efficacy, leading to longer breastfeeding durations [36]. Through shared knowledge and community support, these groups empower individuals and inspire many to become advocates or become lactation specialists. Together, these efforts have contributed to a positive shift in breastfeeding practices, providing stronger support and empowerment for NHB women.

### Limitations

NHANES data are self-reported and, thus, recall biases may exist since this study was not designed to surveil all breastfeeding practices. Further, social desirability may skew self-report data; undesirable behaviors may have been underreported during the interview. Additionally, interviewer bias is a concern, as household interviews are conducted as part of NHANES. Another key limitation is the lack of information on why some mothers never initiated breastfeeding or stopped early. This is significant because many mothers and infants face physiological barriers to breastfeeding. For instance, infants with a cleft palate may struggle to breastfeed due to their inability to create the negative pressure needed for effective latching and the lack of a hard surface to press against the nipple to stimulate milk flow [37]. Infant conditions like galactosemia can make breastfeeding unsafe, as consuming sugars from breastmilk can trigger a serious, potentially-life threatening disease with multiorgan involvement [38]. On the maternal side, health issues such as inflammatory and infectious processes, persistent pain, dermatologic conditions, and disorders of lactogenesis often require medical care and can interfere with successful breastfeeding [39]. Beyond health-related barriers, factors such as social norms may also influence a mother’s decision to initiate or continue breastfeeding. Data on these factors could provide valuable insights for developing research-informed efforts for improving breastfeeding rates. In line with this, there are confounders for which the current study did not account. For example, a mother’s employment has been associated with breastfeeding duration; mothers working in service/labor jobs tend to have the lowest breastfeeding duration compared to women in managerial positions and who were not working; Whitley et al. (2021) hypothesize this is due to it being less feasible for breastfeeding to take place. There are other confounding variables for which we cannot account given the dataset [40]. Given the limited alternative data available, NHANES is still one of the best-quality sets of data for breastfeeding trend analyses.

NHANES data regarding breastfeeding included data from March 2020. During this time, the COVID-19 pandemic received national attention and led to shifts in various facets of life. Although pre-pandemic historical trends demonstrate longstanding BF disparities prior to COVID-19 and 2022 formula shortages in the United States, hospital practices supportive of breastfeeding suffered with decreased in-person lactation support and decreased hospitalization length alongside the pandemic [41]. Therefore, breastfeeding rates may have been influenced by the pandemic.

## 5. Conclusions

Without considering social and behavioral context, it may appear that breastfeeding is a mother’s choice. However, women living in the United States, particularly NHB women, face structural inequities and barriers that make “choosing” to breastfeed an illusion. Achieving an equitable playing field for all women requires addressing structural barriers and the lack of culturally tailored interventions. These structural barriers include limited access to paid maternity leave, insufficient access to breastfeeding education, and widespread disparities in healthcare quality and support. Before individual preferences can be considered, there needs to be an equitable landscape for women to be able to even consider breastfeeding. Economic and structural barriers have long existed, and the recent infant formula shortage has only made these gaps in the system more apparent. The idea that breastfeeding is free of cost is a fallacy. Breastfeeding is only free if milk is produced and if the baby latches well. Beyond that, it is only free if the mother has the ability to be in the baby’s presence “on demand” and/or is able to express breastmilk that keeps up with the baby’s feeding schedule, is able to afford quality foods for herself that support the production of breastmilk, is able to afford clothing, nipple creams, and/or nursing pads that support lactating breasts, does not have medical complications, has access to lactation support, and is supported in her ability to pump breastmilk at work, if applicable. Specifically, access to lactation consultants has been shown to play a crucial role in improving breastfeeding rates [42]. Programs like the Breastfeeding Heritage and Pride (BHP) initiative, designed by the Hispanic Health Council, demonstrate the importance of access to culturally tailored lactation support. The BHP program specifically addresses barriers identified such as a lack of role models, embarrassment about breastfeeding in public, limited social support and breastfeeding knowledge, and the norm of formula feeding [43]. These findings highlight the need not only for more lactation consultants but also for training in culturally congruent care to better support diverse communities and address persistent breastfeeding disparities.

Lactation support and breastfeeding support as mothers return to work must be culturally informed. Strategies include culturally responsive interventions that address social norms unique to NHB women. For example, breastfeeding courses are already affinity spaces (i.e., courses for pregnant individuals), but most courses may not tackle the unique experiences of NHB women and other women of color. Offering culturally tailored breastfeeding courses for NHB women to increase affinity and a sense of belonging is recommended. Breastfeeding education also needs to begin during the prenatal period. These courses could be offered outside of the hospital setting, in comfortable spaces where NHB women can connect with other NHB women and be provided with basic education related to breastfeeding, but also education about the historical legacies about NHB women breastfeeding that have perpetuated the stigma in this community.

For women eligible for WIC support, there needs to be a balanced message related to breastfeeding and formula. For women who freely choose to not breastfeed, the offering of formula may come later rather than prior to the birth of the child. And for women who are ineligible for WIC support, the costs of formula should be discussed as well as possible issues related to the access and availability of formula if the child has allergies or if there are larger societal issues, e.g., supply chain, bacterial contamination. In addition, there continue to be inequities in structural supports despite the Patient Protection and Affordable Care Act (2010). Proper lactation space and suitability, mandated coverage for all types of employees, and a requirement of all types of employers to develop lactation policies are also needed [44]. The development of equitable policy can foster self-empowerment and self-reliance and, as a result, promote the freedom to choose breastfeeding. While policy-level changes may take longer than 40 weeks, the power of community-level, culturally responsive interventions at local, regional, and national levels should not be underestimated. Healthy People 2030 commits to increasing the proportion of infants breastfed for one year from 35.9% (2015) to 54.1% [10].

## Figures and Tables

**Table 1:** Descriptive characteristics for children aged 0 to 6 years and their mothers among NHANES participants, years 1999 through 2020. Total of 19,830 NHANES respondents, representing 27,883,389 children.

	Ever Breastfed (N = 13,762)	Never Breastfed (N = 6068)	*p* Value
	Presented as N (Weighted %) or Mean (SD)		
Child sex at birth (*n* = 19,830)			
Female	6754 (48.6)	2953 (48.9)	0.5233
Male	7008 (51.4)	3115 (51.1)	
Child Race/Ethnicity (*n* = 19,830)			
Mexican	4053 (16.7)	1244 (13.1)	<0.0001
Other Hispanic	1364 (8.6)	449 (7.2)	
NHW	4395 (55.4)	1644 (49.5)	
NHB	2435 (9.9)	2278 (23.4)	
Other or Multiracial	1515 (9.4)	453 (6.8)	
Child Birth Weight (*n* = 19,624)	7.34 ± 0.02	7.05 ± 0.03	<0.0001
Length of Time Breastfed (*n* = 13,505)			
0–6 Weeks	4387 (25.9)	–	–
>6 Weeks to 6 Months	4885 (35.2)	–	–
>6 Months to 1 Year	2773 (23.9)	–	–
>1 Year	1460 (13.8)	–	
Missing	257 (1.3)		–
Mother’s Age When Born (*n* = 19,758)	28.4 ± 0.11	26.0 ± 0.15	<0.0001
Mother Smoked While Pregnant (*n* = 19,768)			
Yes	1161 (9.2)	1266 (23.6)	<0.0001
No	12,581 (90.7)	4760 (75.4)	
Missing	20 (0.1)	42 (1.0)	
Mother’s US Citizenship (*n* = 17,195)			
Yes	11,430 (81.7)	5474 (90.2)	<0.0001
No	257 (1.5)	34 (0.5)	
Missing	2075 (16.8)	560 (9.3)	
Family Income to Poverty Ratio (*n* = 18,137)	2.52 ± 0.04	1.82 ± 0.04	<0.0001
Mother’s Insurance Coverage (*n* = 19,714)			
Yes	12,453 (92.0)	5542 (91.4)	0.11
No	1240 (7.5)	479 (7.6)	
Missing	69 (0.51)	47 (0.97)	
Household Food Security (*n* = 19,265)			
Full Food Security	8225 (68.3)	3332 (61.4)	<0.0001
Marginal Food Security	1885 (11.1)	961 (13.2)	
Low Food Security	2390 (12.8)	1134 (16.0)	
Very Low Food Security	870 (4.8)	468 (6.4)	
Missing	392 (3.0)	173 (3.0)	
Mother’s General Health Condition (*n* = 19,828)			
Excellent	7779 (60.0)	3142 (52.0)	<0.0001
Very Good	3195 (23.4)	1431 (25.1)	
Good	2290 (13.7)	1203 (18.5)	
Fair	470 (2.7)	258 (3.8)	
Poor	27 (0.2)	33 (0.6)	
Missing	1 (0.0)	1 (0.0)	
Mother Seen Mental Health Professional in Past Year (*n* = 6571)			
Yes	169 (1.7)	119 (2.6)	<0.0001
No	4142 (39.9)	2141 (44.0)	
Missing	9451 (58.4)	3808 (53.3)	

*p* value determined from Rao–Scott chi-square (categorical variables) and F-tests from weighted regression (continuous variables). Model 1: Adjusted for mother’s age when born. Model 2: Additionally adjusted for PIR. Model 3: Additionally adjusted for citizenship status, insurance coverage, and food security category, for mother smoking while pregnant.

**Table 2:** Association between race/ethnicity and other sociodemographic factors on prevalence of ever being breastfed among 19,830 NHANES participants, an estimated 27,942,236 US 0–6-year-olds from 1999 to 2020. Presented as prevalence ratios (PRs) and associated 95% confidence intervals (CIs).

	No. Breastfed (Weighted %)	Crude	Model 1	Model 2	Model 3
Race-Ethnicity					
NHW	4395 (74.3)	1.00 (Referent)	1.00 (Referent)	1.00 (Referent)	1.00 (Referent)
NHB	2435 (52.3)	0.80 (0.79–0.82)	0.84 (0.82–0.85)	0.87 (0.85–0.88)	0.84 (0.82–0.86)
Mexican	4053 (76.6)	1.02 (1.01–1.04)	1.06 (1.04–1.07)	1.10 (1.08–1.12)	1.05 (1.03–1.07)
Other Hispanic	1364 (75.3)	1.01 (0.98–1.04)	1.03 (1.00–1.06)	1.06 (1.04–1.09)	1.02 (0.99–1.04)
Other/Multi	1515 (78.1)	1.04 (1.01–1.07)	1.04 (1.01–1.07)	1.05 (1.02–1.08)	1.03 (1.00–1.06)
Mother’s Age at Birth					
20 and under	1888 (55.0)	0.78 (0.76–0.80)	0.80 (0.77–0.82)	0.84 (0.81–0.86)	0.86 (0.83–0.88)
21–25	3453 (65.9)	0.87 (0.85–0.89)	0.88 (0.86–0.90)	0.91 (0.89–0.93)	0.93 (0.91–0.95)
26–30	3892 (77.5)	0.98 (0.96–1.00)	0.98 (0.96–1.00)	0.99 (0.97–1.02)	1.00 (0.98–1.02)
31–35	3005 (79.7)	1.00 (Referent)	1.00 (Referent)	1.00 (Referent)	1.00 (Referent)
36 and older	1510 (79.2)	0.99 (0.97–1.02)	0.99 (0.97–1.02)	1.00 (0.97–1.02)	1.00 (0.97–1.02)
Poverty-to-Income Ratio					
<1	4217 (61.5)	0.81 (0.80–0.83)	–	0.86 (0.84–0.88)	0.88 (0.86–0.91)
1–2	3287 (68.2)	0.87 (0.85–0.89)	–	0.90 (0.88–0.92)	0.92 (0.90–0.94)
2–3	1734 (76.0)	0.94 (0.92–0.96)	–	0.96 (0.94–0.99)	0.97 (0.95–1.00)
3+	3349 (61.5)	1.00 (Referent)	–	1.00 (Referent)	1.00 (Referent)
Mother’s Insurance Coverage					1.01 (0.98–1.04)
Yes	12,453 (72.2)	1.00 (Referent)	–	–	1.00 (Referent)
No	1240 (71.7)	0.99 (0.97–1.03)	–	–	1.01 (0.98–1.04)
Mother’s US Citizenship					
US Citizenship	11,430 (70.0)	0.84 (0.79–0.88)	–	–	0.86 (0.81–0.91)
Non-US Citizenship	257 (87.7)	1.00 (Referent)	–	–	1.00 (Referent)
Food Security					
Full	8225 (74.1)	1.00 (Referent)	–	–	1.00 (Referent)
Marginal	1885 (68.3)	0.94 (0.92–0.97)	–	–	1.02 (0.99–1.05)
Low	2390 (67.4)	0.93 (0.91–0.96)	–	–	1.01 (0.99–1.04)
Very Low	870 (65.7)	0.92 (0.89–0.95)	–	–	1.02 (0.99–1.06)
Mother Smoked While Pregnant					
No	12,581 (75.6)	1.00 (Referent)	–	–	1.00 (Referent)
Yes	1161 (50.2)	0.78 (0.76–0.80)	–	–	0.82 (0.79–0.84)

Model 1: Adjusted for mother’s age when born. Model 2: Additionally adjusted for PIR. Model 3: Additionally adjusted for citizenship status, insurance coverage, and food security category, for mother smoking while pregnant.

## Data Availability

The datasets generated or analyzed during the current study are available in the Centers for Disease Control and Prevention National Health and Nutrition Examination Survey (NHANES) database, https://wwwn.cdc.gov/nchs/nhanes/ (accessed on 12 February 2025).

## Supplementary Materials

The following supporting information can be downloaded at: https://www.mdpi.com/article/10.3390/ijerph22030428/s1, Table S1: Percentage of infants ever breastfed, stratified by duration breastfed in NHANES cohort: United States, 1999–2020; Table S2: Percentage of infants who were breastfed by NHANES cohort and race–ethnicity: United States 1999–2020; Table S3: Average number of days breastfed by race-ethnicity: United States, 1999–2020; Table S4: Percentage of infants who were ever breastfed by PIR and race–ethnicity: United States, 1999–2020.
