# Supplementary material for: Examining Associations Between Sociodemographic Characteristics and Ever Breastfed Children, NHANES 1999–2020
Source: Int J Environ Res Public Health. 2025 Mar 14;22(3):428. doi: 10.3390/ijerph22030428 (PMC11942616; doi:10.3390/ijerph22030428)
Supplement: Supplementary file 1 [file ijerph-22-00428-s001.zip › ijerph-3169224-supplementary.pdf]

Supplemental Table S1: Percentage of infants ever breastfed, stratified by duration breastfed in NHANES cohort: United States, 1999–2020 (See Figure 2).

	0–6 weeks		> 6 weeks to 6 months		> 6 months to 1 year		> 1 year		Ever Breastfed	
	(%)	Std	(%)	Std	(%)	Std	(%)	Std	(%)	Std
1999–2000	27.7	2.5	41.8	2.2	20.9	1.9	9.7	1.7	62.1	3.2
2001–2002	24.6	1.1	38.0	1.7	27.9	1.5	9.5	1.4	65.2	3.0
2003–2004	25.8	3.1	40.1	2.1	22.8	2.7	11.3	2.0	64.0	3.2
2005–2006	25.6	1.9	35.2	1.0	26.1	2.2	13.1	1.4	71.5	2.1
2007–2008	28.9	1.5	36.5	2.4	23.0	1.8	11.6	1.8	69.2	2.5
2009–2010	28.2	1.6	36.2	1.6	24.0	1.8	11.6	1.0	70.9	1.1
2011–2012	26.6	2.1	32.6	1.4	25.2	1.8	15.6	1.4	75.1	1.8
2013–2014	27.3	1.2	37.0	2.0	23.9	2.6	11.8	1.3	75.8	1.8
2015–2016	25.6	1.1	32.9	2.4	24.0	1.9	17.5	2.0	78.0	2.5
2017–2020	24.0	1.1	31.6	2.1	23.7	1.4	20.6	1.6	82.4	1.1

Supplemental Table S2: Percentage of infants who were breastfed by race-ethnicity: NHANES cohort United States, 1999–2020 (See Figure 3).

	Mexican		Other Hispanic		White		Black		Other/Multi		Overall	
	(%)	Std	(%)	Std	(%)	Std	(%)	Std	(%)	Std	(%)	Std
1999–2000	70.9	3.7	72.0	4.4	65.8	5.0	37.1	2.7	69.0	5.9	62.1	3.2
2001–2002	73.0	2.2	68.6	8.0	67.4	3.2	44.0	5.9	72.6	6.0	65.2	3.0
2003–2004	73.3	4.2	63.3	7.6	67.7	4.4	37.7	3.7	66.5	5.9	64.0	3.2
2005–2006	77.1	2.6	75.5	6.3	73.0	2.4	55.9	4.5	73.0	7.7	71.5	2.1
2007–2008	75.4	1.6	70.0	3.8	70.6	4.4	51.8	5.3	78.0	4.0	69.2	2.5
2009–2010	72.6	3.9	70.7	6.3	73.5	1.7	54.5	3.4	76.7	3.6	70.9	1.1
2011–2012	73.8	3.2	82.1	3.9	79.5	2.2	54.0	5.9	81.4	3.1	75.1	1.8
2013–2014	81.9	2.0	77.8	4.8	81.2	2.2	50.9	3.6	71.4	4.7	75.8	1.8
2015–2016	78.5	2.4	73.9	3.0	82.5	2.5	58.2	4.8	84.3	1.8	78.0	2.5
2017–2020	85.3	2.3	83.4	2.8	83.2	1.5	69.3	3.0	88.7	2.2	82.4	1.1

Supplemental Table S3: Average number of days breastfed by race–ethnicity: NHANES cohort United States, 1999–2020 (See Figure 4).

	Mean days	Std
Mexican	193.8	4.9
Other Hispanic	195.1	8.7
White	210.1	4.9
Black	170.4	5.2
Other/Multi	218.9	8.4

Supplemental Table S4: Percentage of infants who were ever breastfed by PIR and race-ethnicity: NHANES cohort United States, 1999–2020 (See Figure 5).

	PIR ≤ 1		1 < PIR ≤ 2		2 < PIR ≤ 3		PIR > 3	
	(%)	Std	(%)	Std	(%)	Std	(%)	Std
Mexican	75.3	1.3	77.1	1.5	77.7	2.4	79.5	2.9
Other Hispanic	69.8	3.0	75.7	2.5	80.0	3.2	80.3	3.6
White	61.3	2.6	67.0	2.3	77.0	2.0	82.4	1.2
Black	41.9	1.9	54.4	1.7	62.5	2.5	71.1	2.1
Other/Multi	63.1	2.8	70.9	3.7	81.1	3.4	89.6	1.6
